# A research agenda to reinforce rabies control: A qualitative and quantitative prioritization

**DOI:** 10.1371/journal.pntd.0006387

**Published:** 2018-05-04

**Authors:** Anne M. G. Neevel, Tessa Hemrika, Eric Claassen, Linda H. M. van de Burgwal

**Affiliations:** 1 Athena Institute, VU University, Amsterdam, the Netherlands; 2 Viroclinics Biosciences, Rotterdam, the Netherlands; 3 Artemis One Health, Utrecht, the Netherlands; Universidad Nacional Mayor de San Marcos, PERU

## Abstract

**Background:**

Despite the existence of safe and effective vaccines, rabies disease still causes an estimated 59,000 human deaths a year in the endemic areas in Asia and Africa. These numbers reflect severe drawbacks regarding the implementation of PrEP and PEP in endemic settings, such as lack of political will and low priority given to rabies. Since these contextual factors have proven to be persistent, there is an urgency to improve current strategies or develop novel approaches in order to control rabies disease in the future.

**Methods/Findings:**

This study aimed to identify and systematically prioritize the research needs, through interviews and questionnaires with key-opinion-leaders (KOLs). A total of 46 research needs were identified and prioritized. The top research needs are considered very high priority based on both importance for rabies control and need for improvement. KOLs agree that animal rabies control remains most important for rabies control, while research on human host, agent (rabies virus) and the environment should be prioritized in terms of need for improvement. A wide variety in perceptions is observed between and within the disciplines of virology, public health and veterinary health and between KOLs with more versus those with less experience in the field.

**Conclusion/Significance:**

The results of this study give well-defined, prioritized issues that stress the drawbacks that are experienced by KOLs in daily practice. The most important research domains are: 1) cheap and scalable production system for RIG 2) efficacy of dog mass vaccination programs and 3) cheap human vaccines. Addressing these research needs should exist next to and may reinforce current awareness and mass vaccination campaigns. The differences in perspectives between actors revealed in this study are informative for effective execution of the One Health research agenda.

## Author summary

Rabies is a 100% vaccine-preventable disease but invariably fatal once symptoms occur. Annually, tens of thousands of people die after being infected with rabies virus, predominantly through bites or scratches of infected dogs. The stable mortality rates highlight the limitations of current disease specific interventions, including prophylaxes, awareness campaigns and mass vaccination of dogs. Consequently, research is needed to develop improved and novel strategies that circumvent the barriers faced in implementation in endemic settings. Interest for rabies, however, is limited and to effectively allocate budgets the field would benefit from a more focused research agenda. This study prioritized research topics based on the importance for rabies control and the need for improvement. According to experts, research should focus on 1) cheap and scalable production systems for RIG; 2) efficacy of dog mass vaccination programs and; 3) development of a cheap human vaccine. By elucidating differences in perceptions of stakeholders between disciplines and between those with more versus those with less experience in the field, this study also provides practical insights to inform stakeholders concerned with the implementation of interdisciplinary collaboration in the field of rabies. The prioritization of rabies-specific research needs is a vital step in accelerating innovation necessary to decrease the burden of disease.

## Introduction

Rabies is a neglected tropical disease causing an estimated 59,000 human deaths a year [1]. Human rabies is 100% preventable by either pre-exposure prophylaxis (PrEP) or post-exposure prophylaxis (PEP) which together effectively prevent approximately 372,000 deaths yearly [2]. In resource-poor settings, however, these prophylaxes are frequently not accessible, incomplete or delayed and consequently, almost 96% of all human cases occur in Africa and Asia despite the fact that rabies virus circulates worldwide [3]. Without treatment options and effective animal rabies control, human rabies will continue to be a social and economic burden.

An important and cost-effective strategy in the control of human rabies is the prevention of infection. Transmission occurs via saliva of animal reservoirs and dogs are the major (90%) source of infection to humans [4]. Mass vaccination of domestic dogs has resulted in effective control of both canine rabies and human rabies when a coverage ratio of 70% is achieved and maintained [5–7]. Due to the size and rapid turnover of dog populations [8] this requires long-term determination which poses a challenge for most of the developing world, due to e.g. a lack of resources, diagnostic capacity and in-country expertise [4, 9, 10]. Addressing these contextual factors could enable rabies elimination, as was achieved in Latin American and Caribbean countries [5], but is hard to achieve given the low priority given to rabies [4].

In settings with little political commitment, prevention and clinical interventions are more feasible strategies to improve health [11]. For rabies, this can be translated in an urgency to improve current control tools and develop novel strategies for rabies control. However, a focus and direction in research and development in the field of rabies is lacking as literature publicizes different priorities [12–14]. Considering the limited resources available for NTDs and rabies in specific, such direction could accelerate the control of rabies in the future. Therefore, the aim of this research was to assess and prioritize the research needs that could improve current strategies or lead to the development of novel strategies to control rabies disease.

## Methods

For the identification and prioritization of research needs a metrics based key-opinion-leader (KOL) approach was applied as a means to incorporate multiple perspectives, in the form of both qualitative and quantitative data. This triangulation of data is considered suitable for prioritization in health care where topics are often considered too delicate for quantitative prioritization alone and consensus between actors from different disciplines is hard to achieve [15–18]. The KOL approach is applied since patients are hardly accessible in the case of rabies, and inclusion of health sector professionals is considered vital for valorization of health research in general, which further increases the relevance of this approach [19].

### Participant selection

For this study, KOLs were defined as individuals with extensive knowledge in the field of virology, public health and/or veterinary (public) health in the context of rabies. KOLs were identified via a web search on representatives of rabies initiatives and (keynote) speakers at international conferences. Additionally, snowball sampling was employed, which allowed the researchers to approach a global network of rabies experts. To ensure a high level of expertise, rabies experts with at least an MSc degree or sufficient rabies-related work experience (>5 years) were approached to participate in this study.

To ensure data richness, KOLs representing different fields of expertise, contexts of expertise and with different professions were selected for participation in the interviews and questionnaires. As rabies disease is a global problem, international KOLs were selected with a special focus on endemic settings in Africa and Asia. This included rabies experts working for non-profit seeking knowledge institutes, for-profit (pharmaceutical) companies, doctors (MD, DVM), non-governmental organizations (GARC), and regulatory and public health authorities (FAO, WHO, OIE).

### Research framework

To obtain a comprehensive overview of the research required to enable rabies control, the epidemiological triangle was used [20, 21]. This framework covers the components important for disease transmission and consequently the targets for infectious disease control: the agent (rabies virus), human and animal hosts and the environment [21]. Relevant research needs are defined as research that contributes to assaulting virulence of the agent (e.g. antivirals and passive immunization), raising susceptibility of the host (active immunization) and/or diminishing the favorability of the (sociocultural and physical) environment.

### Semi-structured interviews

The multi-staged prioritization process started with the identification of research needs through interviews with KOLs. KOLs were prompted with semi-structured interview questions based on the components of the epidemiological triangle. The interviews pursued questions about the strengths and weaknesses of current strategies, followed by the question what research is needed to advance rabies control in order to prime the respondents to research on both novel strategies and improvements to current strategies. Probing was based on the concepts of the epidemiological triangle. Interviews were conducted by two researchers via phone or Skype and lasted, on average, 30 minutes. Interview invitations were sent until data saturation (no new research needs mentioned in four subsequent interviews) was reached in the interviews.

Data from the interviews was analyzed through thematic coding by two independent researchers, leading to inductively derived research needs [22]. Subsequent discussion led to agreement regarding the final coding of the research needs, thereby making the formulations as clear, complete and concise as possible.

### Online questionnaire

The identified research needs formed the basis for the questionnaire. Research needs that were mentioned by only one KOL were not considered to have priority and were therefore excluded from the questionnaire. The anonymous questionnaire consisted of two parts: ranking of individual research needs and ranking of the components of the epidemiological triangle. During the ranking exercise, participants were asked to apply two criteria to encourage active and balanced prioritization [18]: importance for rabies control and need for improvement. This enabled respondents to distinguish clearly between those aspects that are already in place and research that may have a large impact on the unmet need [16, 17].

The final questionnaire consisted of 30 questions. The questionnaire was pilot tested and distributed through the online web survey program SurveyMonkey. The questionnaire was distributed among 172 KOLs selected through a web search based on abovementioned criteria. This selection included the interview participants. Additionally, KOLs were encouraged to send the questionnaire to colleague rabies experts. For all respondents demographics were checked for compliance with the inclusion criteria. A reminder was sent after 7 and 11 days to increase the response-rate. A copy of the questionnaire is deposited with DANS (see Data Availability Statement).

#### Research prioritization

KOLs were asked to assign low, moderate or high priority to all research needs based on the evaluation criteria ‘importance for rabies control’ and ‘need for improvement’. If KOLs felt that they did not have the expertise on a certain component, they could skip research prioritization of research needs for this particular component. For further analysis, the priority groups were quantified by awarding each high, moderate and low priority with three, two, and one points, respectively. The mean scores ($\bar{x}$) were re-scaled to range from 0 to 100 (instead of 1 to 3) by using the following formula:

$$Score=\frac{\bar{x}-1}{2}*100$$

Although linearity between the scores could not be verified either between or within respondents, the current method was preferred over a need per need comparison due to its simplicity. The method encourages participation of a broad set of stakeholders and therefore offers a comprehensive overview of research priorities. Considering the possible differences in the weight of priority, research needs were presented in priority groups and not scores. To facilitate meaningful interpretation, priority groups were narrowed to differentiate between very low (0–20), low (21–40), moderate (41–60), high (61–80) and very high (81–100) priority. Differences between the need for improvement and importance for rabies control were tested by using the dependent t-test.

#### Component prioritization

Since KOLs might be biased towards certain types of research needs based upon their own expertise, KOLs were asked to rank the components (agent, human host, animal host and environment) in order to check for such overall bias. The components were ranked on the evaluation criterion ‘importance for rabies control’ by distributing 100 points over the different components (more points being more important). To assess whether differences existed in the distribution of points allocated to the individual components between KOLs from different contexts a Friedman’s ANOVA was applied. If a significant difference (p<0.05) was found, a post-hoc Wilcoxon signed rank test was performed and a Bonferroni correction was applied to adjust for multiple testing. Statistical analyses were performed with the statistical program SPSS, version 23.0.

#### Statistical analysis between groups

Next, analyses were performed to look for significant differences between the various groups of respondents. It was hypothesized that a participant’s rabies related experience would influence that person’s perceived research needs. To test this hypothesis, respondents were stratified according to their field expertise, context of experience and years of experience. Differences on points allocated to the importance of the components were tested by means of the Kruskal-Wallis test. If a significant difference (p-value <0.05) was found, a post-hoc Mann-Whitney test was performed and a Bonferroni correction was applied. If a significant difference in component prioritization was found between abovementioned respondent groups, and thus response bias assumed, it was checked whether this effect was reflected in the prioritization of research needs. Scores were corrected for the number of respondents per group and, if needed, assigned priority categories were corrected.

## Results

### Respondent demographics

**Fig 1:**
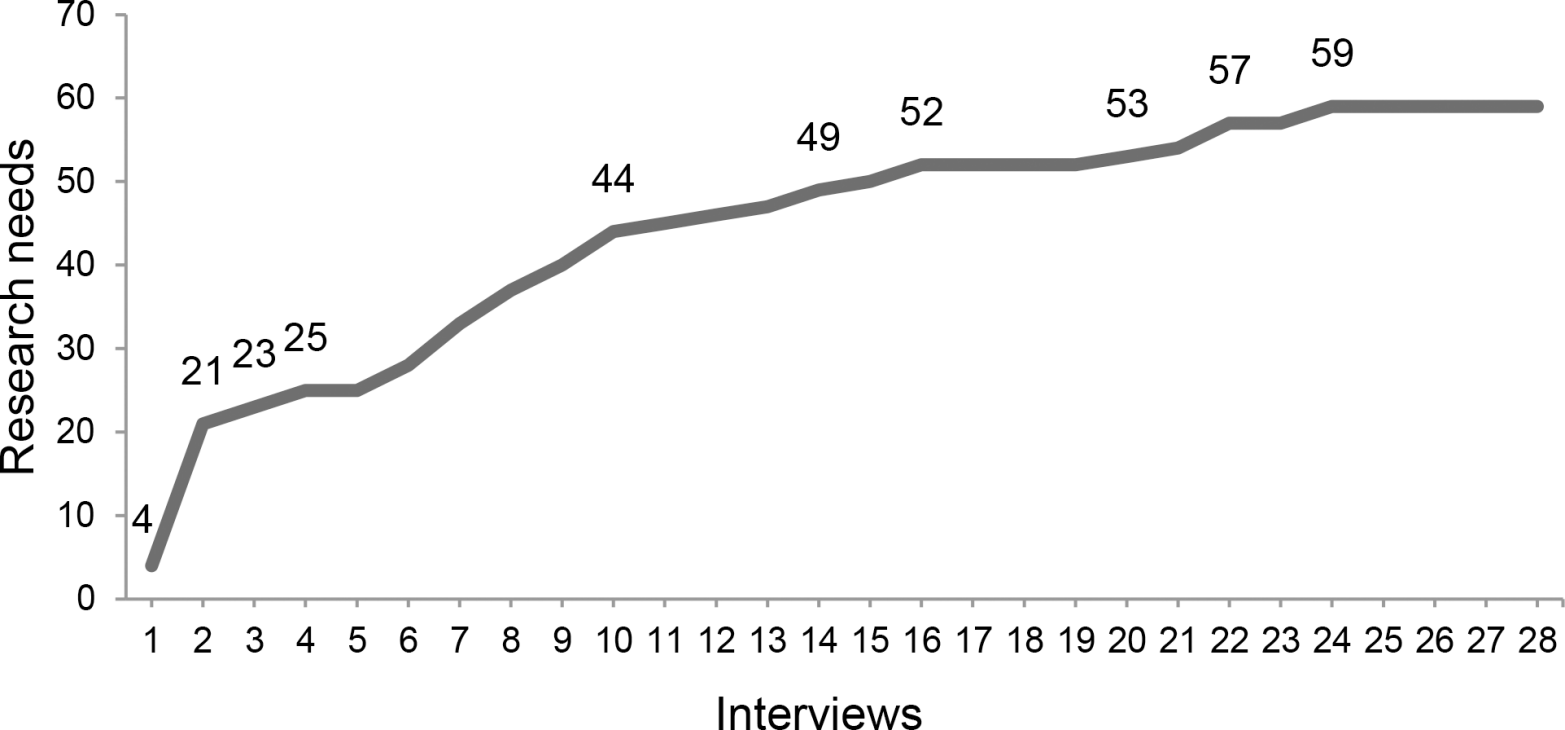
Saturation curve of research needs. Saturation was reached after 24 interviews.

A total of 28 KOLs participated in the interviews, after which data saturation was reached (Fig 1). Other invitees either did not respond (n = 27), did not consider themselves experts based on our definition (n = 8), had no time to participate within the indicated time frame (n = 7) or perceived a conflict of interest (n = 3). A total of 126 (73% of initially invited) participants filled out the questionnaire, of which one response was excluded because the inclusion criteria for KOLs were not met. The age distribution of the KOLs (n = 125) was: 55-up (44%), 40–55 (42%) and 25–40 (14%). The highest obtained academic rank of KOLs are a PhD (39%), followed by professor (30%), Master of Science (27%), and Bachelor of Science (3%). The majority of the KOLs work as scientists (58%), but may also work as policy makers (25%), industry (13%) and medical professionals (30%). The distribution of rabies related expertise is shown in Table 1. Based on these demographic profiles, we consider our KOL sample to have significant expertise in rabies research and representative for the different disciplines.

**Table 1:** Respondent’s rabies related work experience.

		Interviews	Survey^b^
Total (n)		28	125
**Field of expertise**^**a**^	Virology	12 (43%)	53 (42%)
	Public Health	14 (50%)	76 (61%)
	Veterinary (public) health	10 (36%)	66 (53%)
**Context of expertise**	Low- and middle income countries	4 (14%)	65 (52%)
	High income countries	12 (43%)	25 (20%)
	Both	12 (43%)	35 (28%)
**Years of experience**	0–5	3 (11%)	14 (11%)
	5–10	4 (14%)	22 (18%)
	10-up	21 (75%)	89 (71%)

^a^Participants could be active in more than one field of expertise: 73 indicated to have expertise in one field, 34 in two, 18 in three.

^b^Participants of the interviews also received the survey, which was filled out anonymously, therefore percentages were calculated on the basis of n = 153.

### Identified research needs

A total of 59 research needs emerged from the interviews, of which 46 were mentioned by more than one KOL. The research needs related to all components of the epidemiological triangle: 10 research needs addressed the animal host, 12 the human host, 13 the rabies virus and 11 the environment.

### Prioritized research needs

**Fig 2:**
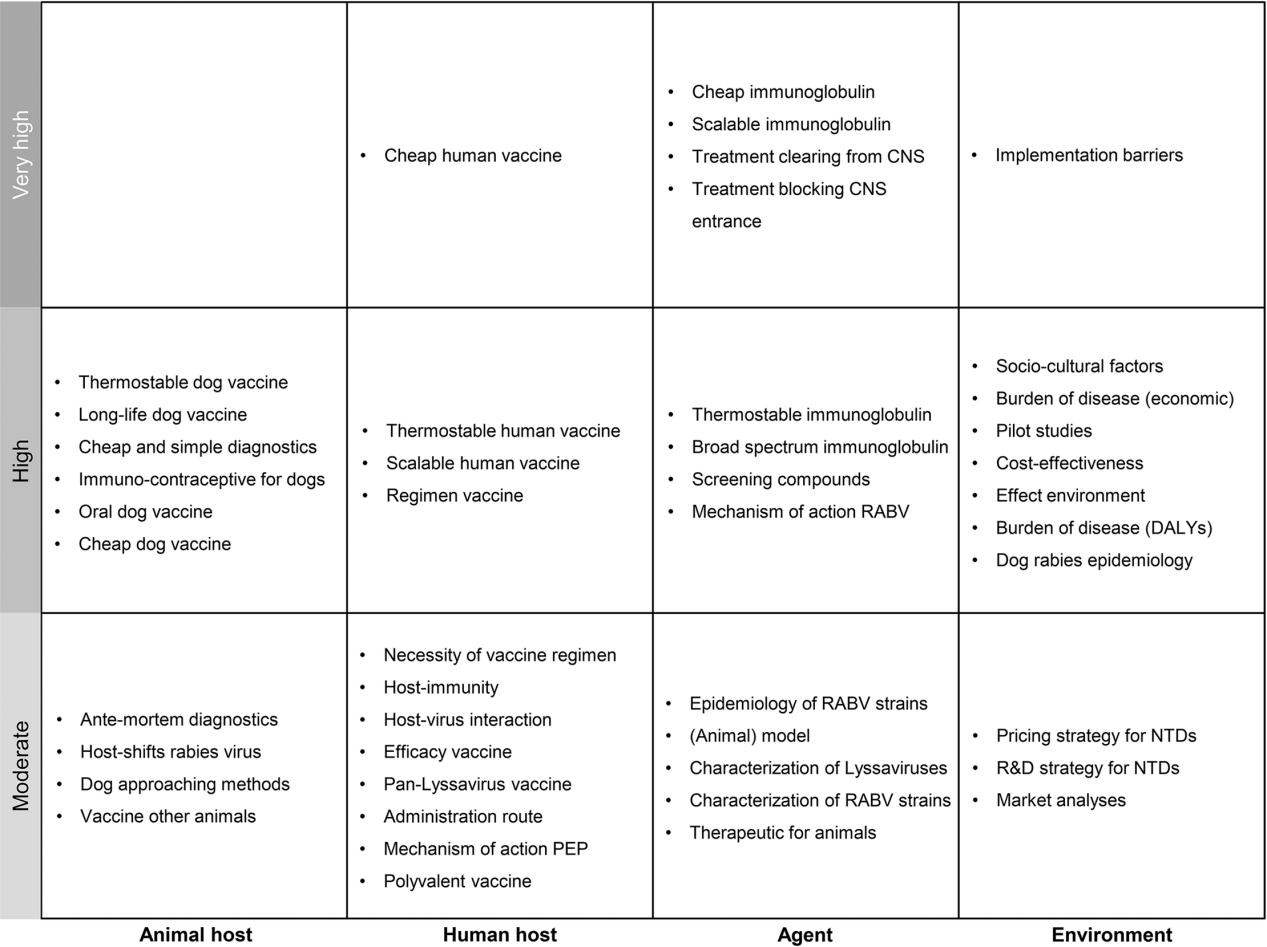
Research needs prioritized for need for improvement. A total of 125 KOLs assessed the research needs of their expertise: animal host (n = 96); human host (n = 88); agent (n = 83); environment (n = 78). Priority groups correspond to scores of 41–60 (moderate), 61–80 (high) and 81–100 (very high). Full descriptions of research priorities and scores can be found in S1 Table. CNS = central nervous system; RABV = rabies virus; R&D = research and development; DALYs = disability adjusted life years; PEP = post-exposure prophylaxes; NTD = neglected tropical disease.

A total of 125 KOLs assessed the research needs of their expertise: animal host (n = 96); human host (n = 88); agent (n = 83) and; environment (n = 78). For all four components, several research needs were assigned to the high priority group. Fig 2 illustrates the average priority (moderate-high-very high) KOLs attributed to the need of improvement of each research need, divided over the different components. For all components, high priority research needs were identified. Very high priority was assigned to research needs linked to the human host, agent and environment. None of the identified research needs was assigned to the very low or low priority group.

**Fig 3:**
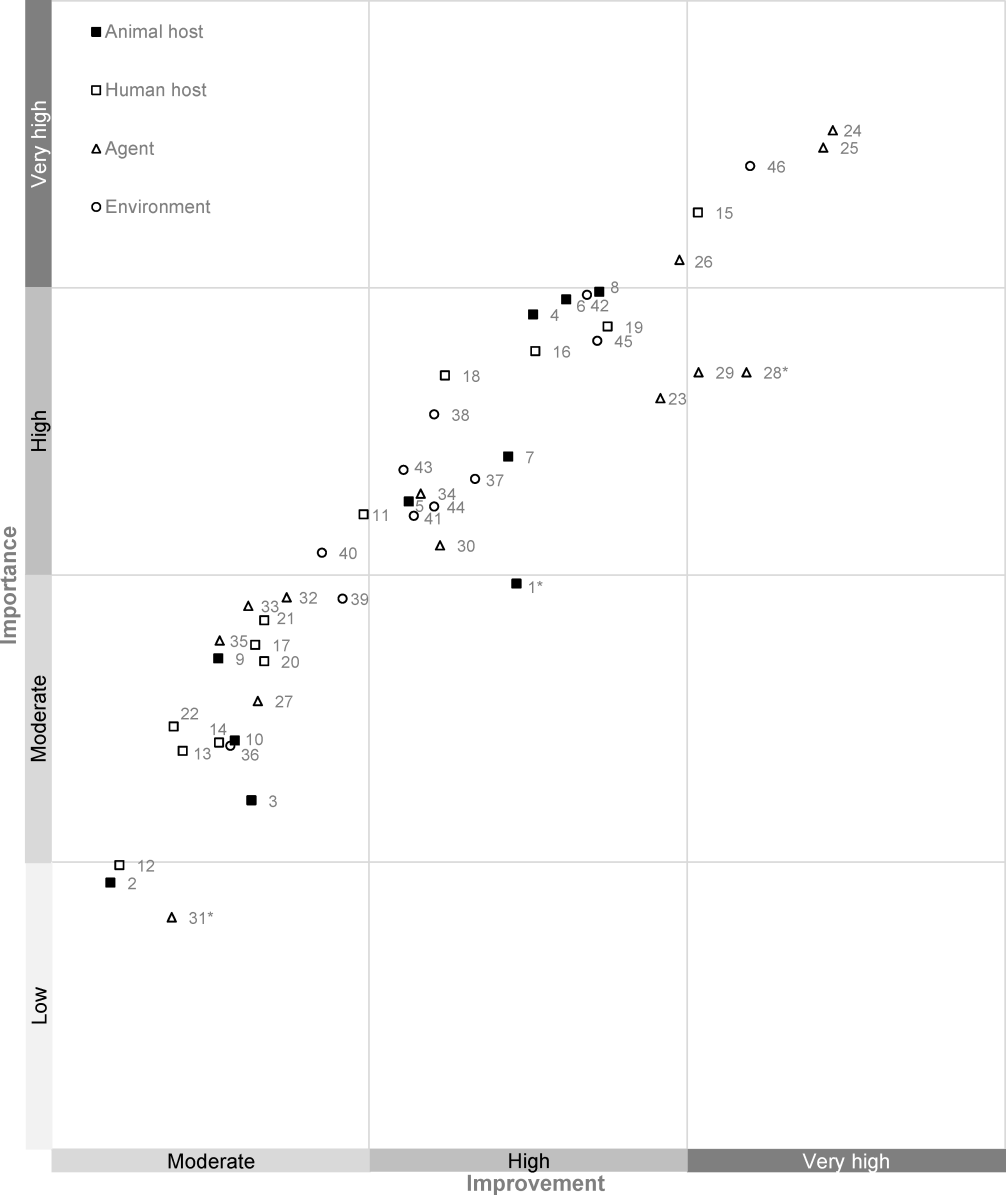
Research needs with high need for improvement are also considered highly important for rabies control. *Significant (p<0.05) difference between the priority group for improvement and importance. Full descriptions of research priorities, corresponding to the numbered value labels, can be found in S1 Table.

In addition, respondents were asked to rank the identified research needs on the importance of that aspect for the control of rabies disease. Based on the combined prioritization of importance and need for improvement (Fig 3), the research needs that should be given very high priority in rabies research, according to KOLs, are 1) developing a cheap alternative for rabies immunoglobulins (RIG), followed by 2) developing an alternative for RIG that is easy to produce, 3) increasing knowledge on factors that hamper the efficacy of dog mass vaccination programs and 4) developing a cheap alternative for the human vaccine.

A comparison of the results reveals modest differences between importance and need for improvement and 11 research needs with significant differences (p<0.05) between the scores of the two criteria (S1 Table). Only 3 out of these research needs could be assigned to different priority groups for importance compared to need for improvement (Fig 3) and they were all given a higher need for improvement than importance for rabies control: the development of treatment options that can clear the virus from the CNS (very high versus high), the development of immuno-contraceptives for dogs (high versus moderate), and the development of treatment for animals (moderate versus low). The other research needs were assigned to the same priority groups for need for improvement and importance.

### Prioritized components

Table 2 shows the ranking of the four components, through distribution of 100 points, with the animal host having the highest mean for importance (37.2), followed by agent (22.1), human (22.0) and environment (19.5) (p<0.001). The component prioritization shows a high standard deviation, indicating a wide variety in the points allocated by the KOLs. The number of points allocated to agent and environment ranged from 0 to 70, for human from 0 to 75, and for animal host from 0 to 100 points. This illustrates that the KOLs have divergent views on the importance and need for improvement of the components for rabies control.

**Table 2:** Component prioritization. Although there is a high standard deviation, tackling rabies in the animal host is considered most important for rabies control. LMIC = low- and middle income countries; HIC = high income countries.

	Agent		Human host		Animal host		Environment	
	Mean	SD	Mean	SD	Mean	SD	Mean	SD
Overall	22,1	14,0	22,0	12,5	37,7*	18,2	19,5	15,5
**Field of expertise**								
Public Health	21,4	12,2	29,4*	13,2	30,9	15,9	18,3	15,7
Veterinary (public) health	18,3	12,7	18,8	12,0	36,1	17,6	26,8	20,4
Virology	25,7	18,8	22,7	12,2	38,7	13,7	13,0	8,6
Multiple	23,1	13,6	18,3	10,3	42,6	20,7	19,0	13,3
**Context of expertise**								
LMIC	19,3	12,8	22,4	14,4	36,6	19,4	21,6	17,3
HIC	26,5	16,1	23,8	11,1	34,1	12,7	15,6	12,6
Both	24,2	14,2	19,8	9,2	41,6	19,1	18,1	13,4
**Years of experience**								
0–5	18,5	14,5	22,5	14,4	36,6	19,3	21,6	17,3
5–10	15,1	10,3	23,8	11,1	34,1	12,7	15,6	12,6
10-up	24,5*	14,2	19,8	9,2	41,6	19,1	18,1	13,4

*Significant at P<0.05.

#### Statistical analysis between groups

No significant differences were observed between the different contexts of experience in the component prioritization (Table 2). Conversely, with regards to points allocated to the importance of the human host component, a statistically significant difference between the different fields of expertise was observed. Participants with expertise in public health allocated significantly more points to human host than participants with other or multiple areas of expertise (p = 0.002). Differences were also found across groups with different years of experience for points allocated to the agent component. Participants with more than 10 years of work experience related to rabies allocated significantly more points to the importance of the agent component than participants with less experience (p = .005).

Based on the results shown in Table 2, it is relevant to look at the effect of the preferred components on the prioritization of research needs. S2 Table shows the research priorities as presented in Fig 2, adjusted for the number of participants (Table 1) in the groups with different field of expertise (for human host) and years of experience (for agent). The table shows that a groups’ preference for a specific component does not affect its prioritization of separate research needs.

## Discussion

This paper provides a unique new dataset in canvassing and prioritizing research needs in rabies going further than mere control of the animal reservoir. KOLs assigned high or very high priority on need for improvement to a total of 26 research needs, and their urgency is amplified by the finding that these research needs are equal to the research needs that were considered important for rabies control (Fig 3). Research on the animal host is considered most important for rabies control, but top priorities reflect the invariable demand for improved preventive and therapeutic strategies for human application to decrease the burden of rabies disease on the short term. Taking into account the limited resources available, research efforts should focus on the research needs that are prioritized as highly important by KOLs, which could be reduced to the following domains: 1) cheap and scalable production systems for RIG; 2) efficacy of dog mass vaccination programs and; 3) cheap human vaccines.

Importantly, literature shows that these unmet needs are addressable. Recent developments regarding production systems for cheap and scalable alternatives for RIG include monoclonal antibodies [23] and nanobodies [24]. The identification of barriers hampering the efficacy of current mass vaccination programs would require such programs to include a qualitative causal component. The few studies that report on barriers to low vaccination coverage show that the collection of these data can lead to the formulation of program-specific strategies to increase vaccination coverage [25–27]. Lastly, novel vaccines using adjuvants have shown encouraging clinical outcomes [28]. The costs of adjuvanted vaccines may exceed the costs of existing vaccines, however, the improved immunogenicity may reduce the total costs of PEP via reduction of doses and number of hospital visits. The characteristics of above-mentioned products, such as costs, scalability and regimen, would improve the accessibility of products in endemic countries of Asia and Africa and, hence, significantly decrease the burden of rabies disease [29].

It can be argued that addressing the research needs presented here could align stakeholders towards effective use and implementation of rabies control programs. Individuals at risk will be more likely to translate awareness in demand [29], willingness to pay [30] and compliance [31], if PrEP and PEP could be obtained and used more easily. The introduction of improved and novel products may, thus, increase the impact of education and awareness programs. Likewise, accurate data on the societal and economic burden of disease could increase political will and advocate funding for rabies control, which is currently suffering from low priority and a lack of resources [32]. Tackling the research needs presented here could, thus, introduce a mutually reinforcing cycle and accelerate the control of rabies disease.

Besides sketching the contents of the rabies research agenda, the current study highlights differences between KOLs that should be used to inform its implementation. The control of rabies, like other zoonoses with a serious socioeconomic impact, could benefit from an One Health approach in which human, animal and environmental health are integrated [33]. This could be hampered by the observed variety in perception on the importance of different epidemiological components (Table 2). The variety between KOLs of different disciplines is not surprising considering the general preference of animal rabies control measures, which seems to have caused a protective tendency by the public health sector. The significant importance that more experienced KOLs (>10 years of experience) give to the agent indicates that the limited successes in the past led to disbelief in possible solutions without a better understanding of the pathogenesis of rabies virus [34]. This finding not only argues for the reintroduction of basic research on research agendas, which has been diminished by ‘impact assessments’ [35], it also informs funders to shift their expectations for the development of novel medications against rabies from short-term to medium-long term. Overcoming these innovation barriers, that can stop research (funding) in a premature phase [34], should coincide with the implementation of research priorities to optimize societal impact.

Despite the differences in KOL’s perceptions, KOLs seem to acknowledge the importance of an One Health approach. It was observed that research needs for all the components were given high priority (Fig 2) and that all components received a significant number of points (Table 2). Taken together, stakeholders are encouraged to converge their activities relatively to the One Health research agenda presented here, which serves as a uniting tool and may provide the necessary focus to achieve global rabies elimination.

## Supporting information

S1 Table List of research needs. (PDF)

S2 Table Priority groups corrected for unequal group sizes. (PDF)
