# Supplementary material for: A research agenda to reinforce rabies control: A qualitative and quantitative prioritization
Source: PLoS Negl Trop Dis. 2018 May 4;12(5):e0006387. doi: 10.1371/journal.pntd.0006387 (PMC5955568; doi:10.1371/journal.pntd.0006387)
Supplement: S1 Table — (PDF) [file pntd.0006387.s001.pdf]

S1 Table. List of research needs. RIG = rabies immunoglobulin; DALY = disability adjusted life year; RABV = rabies virus; PEP = post-exposure prophylaxis; CNS = central nervous system; R&D = research and development; DALYs = disability adjusted life years; NTD = neglected tropical disease. L = low; M = moderate; H = high.

#[a]	Short description[b]	Full description[c]	Importance					Improvement					Δ Mean	Sig. (2-tailed)[d]
			L	M	H	Mean	Score	L	M	H	Mean	Score		
27	(Animal) model	Develop replicable (animal) model for pre-clinical trials	25	31	27	2,02	51	24	30	29	2,06	53	-0,04	0,552
13	Administration route	Improve human vaccine (administration route)	25	42	21	1,95	48	27	37	24	1,97	48	-0,01	0,829
3	Ante-mortem diagnostics	Develop ante-mortem diagnostic test	34	39	23	1,89	44	29	33	34	2,05	53	-0,17	0,013*
9	Approaching methods	Increase knowledge on/method to approach dogs for parenteral vaccination	25	38	33	2,08	54	27	41	28	2,01	51	0,07	0,239
23	Broad spectrum immunoglobulin	Develop alternative for RIG (broad spectrum)	7	32	44	2,45	72	5	26	52	2,57	78	-0,12	0,032*
41	Burden of disease (DALYs)	Increase knowledge on burden of disease (DALYs)	12	32	34	2,28	64	13	32	33	2,26	63	0,03	0,673
42	Burden of disease (economic)	Increase knowledge on burden of disease (economic)	2	28	48	2,59	79	5	31	42	2,47	74	0,12	0,083
33	Characterisation of Lyssaviruses	Increase knowledge on Lyssaviruses (species)	14	42	27	2,16	58	20	39	24	2,05	52	0,11	0,038*
35	Characterisation of RABV strains	Increase knowledge on rabies virus (strains)	17	40	26	2,11	55	21	40	22	2,01	51	0,10	0,073
4	Cheap and simple diagnostics	Improve diagnostic test (cheap and simple)	6	30	60	2,56	78	9	39	48	2,41	70	0,16	0,010*
5	Cheap dog vaccine	Improve dog vaccine (cheaper)	16	35	45	2,30	65	21	30	45	2,25	63	0,05	0,401
15	Cheap human vaccine	Improve human vaccine (cheap)	4	18	66	2,70	85	8	18	62	2,61	81	0,09	0,088
24	Cheap immunoglobulin	Develop alternative for RIG (cheap)	0	15	68	2,82	91	3	12	68	2,78	89	0,04	0,470
38	Cost-effectiveness	Cost-effectiveness study (for animal vaccination)	8	29	41	2,42	71	15	26	37	2,28	64	0,14	0,070
43	Dog rabies epidemiology	Increase knowledge on epidemiology of rabies disease (dog rabies)	11	29	38	2,35	67	17	25	36	2,24	62	0,10	0,103
44	Effect environment	Increase knowledge on epidemiology of rabies disease (effect of environmental factors)	11	33	34	2,29	65	11	34	33	2,28	64	0,01	0,810
17	Efficacy vaccine	Improve human vaccine (efficacy)	24	31	33	2,10	55	28	27	33	2,06	53	0,05	0,496
32	Epidemiology of RABV strains	Increase knowledge on epidemiology of rabies disease (different virus strains)	10	49	24	2,17	58	15	45	23	2,10	55	0,07	0,159
20	Host-immunity	Increase knowledge on host-immunity	18	45	25	2,08	54	22	38	28	2,07	53	0,01	0,863

#[a]	Short description[b]	Full description[c]	Importance					Improvement					Δ Mean	Sig. (2-tailed)[d]
			L	M	H	Mean	Score	L	M	H	Mean	Score		
10	Host-shifts rabies virus	Increase knowledge on host-shifts of rabies virus	29	41	26	1,97	48	26	41	29	2,03	52	-0,06	0,368
21	Host-virus interaction	Increase knowledge on host-virus interaction	19	38	31	2,14	57	26	30	32	2,07	53	0,07	0,306
1	Immuno-contraceptive for dogs	Develop immunocontraceptives for dogs	20	38	38	2,19	59	17	25	54	2,39	69	-0,20	0,000***
46	Implementation barriers	Increase knowledge on factors that hamper the efficacy of current mass vaccination programs	2	14	62	2,77	88	3	19	56	2,68	84	0,09	0,070
6	Long-life dog vaccine	Improve dog vaccine (providing long-life immunity)	8	24	64	2,58	79	13	27	56	2,45	72	0,14	0,002**
36	Market analyses	Conduct market analyses	20	41	17	1,96	48	19	38	21	2,03	51	-0,06	0,388
22	Mechanism of action of PEP	Increase knowledge on mechanism of action of PEP	26	37	25	2,27	49	29	34	25	1,99	48	0,03	0,470
34	Mechanism of action RABV	Increase knowledge on mechanism of action of rabies virus	11	35	37	1,95	66	13	35	35	2,31	63	0,05	0,374
11	Necessity of vaccine regimen	Determine the necessity of current vaccine regimen	16	31	41	2,28	64	20	31	37	2,19	60	0,09	0,240
7	Oral dog vaccine	Improve dog vaccine (oral administration)	15	31	50	2,36	68	12	36	48	2,38	69	-0,01	0,854
14	Pan-Lyssavirus vaccine	Improve human vaccine (pan-Lyssavirus)	25	41	22	1,97	48	26	35	27	2,01	51	-0,05	0,417
37	Pilot studies	Conduct pilot studies to show efficacy of current rabies control programs	13	26	39	2,33	67	15	22	41	2,33	67	0,00	1,000
12	Polyvalent vaccine	Develop polyvalent vaccines (more diseases)	36	34	18	1,80	40	34	30	24	1,89	44	-0,09	0,184
39	Pricing strategy for NTDs	Develop strategy for pricing NTD drugs	13	39	26	2,17	58	13	39	26	2,17	58	0,00	1,000
40	R&D strategy NTD	Develop strategy for R&D of NTD drugs	12	36	30	2,23	62	16	35	27	2,14	57	0,09	0,109
18	Regimen vaccine	Improve human vaccine (regimen)	9	28	51	2,48	74	15	32	41	2,30	65	0,18	0,001**
16	Scalable human vaccine	Improve human vaccine (easy to produce)	11	21	56	2,51	76	14	24	50	2,41	70	0,10	0,060
25	Scalable immunoglobulin	Develop alternative for RIG (easy to produce/ up-scalable)	0	17	66	2,80	90	2	15	66	2,77	89	0,02	0,672
30	Screening compounds	Develop treatment (applying knowledge from other diseases and existing compounds)	15	33	35	2,24	62	14	31	38	2,29	64	-0,05	0,483
45	Socio-cultural factors	Increase knowledge on epidemiology of rabies disease (effect of socio-cultural aspects)	4	29	45	2,53	76	5	30	43	2,68	74	-0,15	0,022*
8	Thermostable dog vaccine	Improve dog vaccine (thermostable)	4	31	61	2,59	80	8	33	55	2,49	74	0,10	0,049*
26	Thermostable immunoglobulin	Develop alternative for RIG (thermostable)	3	24	56	2,64	82	5	24	54	2,59	80	0,05	0,397
19	Thermostable vaccine	Improve human vaccine (thermostable)	7	26	55	2,55	77	8	28	52	2,50	75	0,05	0,417

#[a]	Short description[b]	Full description[c]	Importance					Improvement					Δ Mean	Sig. (2-tailed)[d]
			L	M	H	Mean	Score	L	M	H	Mean	Score		
29	Treatment blocking CNS entrance	Develop treatment (preventing the virus from entering the CNS)	10	23	50	2,48	74	10	12	61	2,61	81	-0,13	0,063
28	Treatment clearing from CNS	Develop treatment (entering and clearing the virus from CNS)	10	23	50	2,48	74	8	11	64	2,67	84	-0,19	0,004**
31	Treatment for animals	Develop treatment for animals	36	34	13	1,72	36	30	27	26	1,95	48	-0,23	0,007**
2	Vaccine other animals	Develop vaccine for animals for which no medical interventions are available yet	44	30	22	1,77	39	31	46	19	1,88	44	-0,10	0,123

[a]Number as provided in Fig 3.

[b]Description as provided in Fig 2.

[c]Description as provided in survey.

[d]Statistical analyses were performed on raw data (before rescaling to 0-100 scale). *,**,*** significance at 0.1, 0.05 and 0.01 levels, respectively.
