# Supplementary material for: A research agenda to reinforce rabies control: A qualitative and quantitative prioritization
Source: PLoS Negl Trop Dis. 2018 May 4;12(5):e0006387. doi: 10.1371/journal.pntd.0006387 (PMC5955568; doi:10.1371/journal.pntd.0006387)
Supplement: S2 Table — (PDF) [file pntd.0006387.s002.pdf]

S2 Table. Priority groups corrected for unequal group sizes. Statistical analyses on component prioritisation lead to the assumption that scores in blue would be higher than average and, thus, new categories would be lower. Priority group* is different from the original category, but not in line with the expectations based on the statistical analyses. CNS = central nervous system; RABV = rabies virus; PEP = post-exposure prophylaxis.

Years of experience - agent (need for improvement)									
**RN (short)**	**Priority group**	**Overall mean (n=83)**	**Mean 0-5 yrs (n=10)**	**Mean 5-10 yrs (n=13)**	**Mean 10-up (n=60)**		**Corrected Mean**	**Corrected score**	**New priority group**
Broad spectrum immunoglobulin	high	2,57	2,50	2,62	2,57		2,56	78,03	high
Cheap immunoglobulin	very high	2,78	2,80	2,92	2,75		2,82	91,22	very high
Scalable immunoglobulin	very high	2,77	2,80	2,92	2,73		2,82	90,94	very high
Thermostable immunoglobulin	high	2,59	2,60	2,92	2,52		2,68	84,00	very high*
(Animal) model	moderate	2,06	1,90	2,15	2,07		2,04	52,01	moderate
Treatment clearing from CNS	very high	2,67	2,80	2,92	2,60		2,77	88,72	very high
Treatment blocking CNS entrance	very high	2,61	2,70	2,77	2,57		2,68	83,93	very high
Screening compounds	high	2,29	2,30	2,62	2,22		2,38	68,87	high
Treatment for animals	moderate	1,95	1,90	2,31	1,88		2,03	51,52	moderate
Epidemiology of RABV strains	moderate	2,10	2,50	2,08	2,03		2,20	60,17	high*
Characterisation of Lyssaviruses	moderate	2,05	2,40	1,77	2,05		2,07	53,65	moderate
Mechanism of action RABV	high	2,27	2,30	2,23	2,27		2,27	63,29	high
Characterisation of RABV strains	moderate	2,01	2,20	2,08	1,97		2,08	54,06	moderate

Field of expertise - human host (need for improvement)									
**RN (short)**	**Priority group**	**Overall mean (n=88)**	**Mean Public Health (n=33)**	**Mean Veterinary (public) health (n=26)**	**Mean Virology (n=17)**	**Mean Multiple (n=49)**	**Corrected Mean**	**Corrected score**	**New priority group**
Necessity of vaccine regimen	moderate	2,19	2,27	2,25	1,92	2,21	2,16	58,08	moderate
Polyvalent vaccine	moderate	1,89	1,73	2,08	1,92	1,92	1,91	45,65	moderate
Administration route	moderate	1,97	2,00	1,75	1,83	2,05	1,91	45,45	moderate
Pan-Lyssavirus vaccine	moderate	2,01	2,00	2,08	2,00	2,00	2,02	51,04	moderate
Cheap human vaccine	very high	2,61	2,65	2,83	2,67	2,50	2,66	83,17	very high
Upscalable human vaccine	high	2,41	2,54	2,58	2,42	2,26	2,45	72,52	high
Efficacy vaccine	moderate	2,06	2,19	1,83	2,42	1,92	2,09	54,54	moderate
Regimen vaccine	high	2,30	2,38	2,08	2,25	2,32	2,26	62,92	high
Thermostable vaccine	high	2,50	2,35	2,67	2,58	2,53	2,53	76,53	high
Host-immunity	moderate	2,07	2,08	1,92	2,42	2,00	2,10	55,13	moderate
Host-virus interaction	moderate	2,07	2,04	2,08	2,17	2,05	2,09	54,26	moderate
Mechanism of action of PEP	moderate	1,95	1,73	2,08	2,00	2,05	1,97	48,33	moderate
